# Electron shuttles enhanced the removal of antibiotics and antibiotic resistance genes in anaerobic systems: A review

**DOI:** 10.3389/fmicb.2022.1004589

**Published:** 2022-09-07

**Authors:** Yuepeng Deng, Kaoming Zhang, Jie Zou, Xiuying Li, Zhu Wang, Chun Hu

**Affiliations:** Institute of Environmental Research at Greater Bay/Key Laboratory for Water Quality and Conservation of the Pearl River Delta, Ministry of Education, Guangzhou University, Guangzhou, China

**Keywords:** anaerobic digestion, antibiotic, antibiotic resistant gene, electron shuttle, biological treatment

## Abstract

The environmental and epidemiological problems caused by antibiotics and antibiotic resistance genes have attracted a lot of attention. The use of electron shuttles based on enhanced extracellular electron transfer for anaerobic biological treatment to remove widespread antibiotics and antibiotic resistance genes efficiently from wastewater or organic solid waste is a promising technology. This paper reviewed the development of electron shuttles, described the mechanism of action of different electron shuttles and the application of enhanced anaerobic biotreatment with electron shuttles for the removal of antibiotics and related genes. Finally, we discussed the current issues and possible future directions of electron shuttle technology.

## Introduction

The discovery of penicillin was one of the greatest milestones in the history of medicine (Zhang et al., 2015). Since then, more and more ingredients with antibacterial and anti-inflammatory effects have been discovered or invented and been given a common name—Antibiotic. Nowadays, antibiotics are widely used in medical, breeding, and livestock fields and the dangers associated with antibiotic abuse are increasingly attracting the attention of researchers (Zhang Q. Q. et al., 2014; Zhang Y. J. et al., 2014). According to statistics, China produces about 210,000 tons of antibiotics every year (Luo et al., 2011), and about 162,000 tons are used in various practices (Chen Y. et al., 2020). It is worth noting that not all antibiotics ingested into the human body or other living organisms are utilized and about 58% of the drug components are excreted from the body directly and thus into the environment (Huang et al., 2020). As a result, both the manufacture and consumption of antibiotics lead to a large amount of antibiotic-containing wastewater into the environment (Zhang et al., 2015; Chen Y. et al., 2020; Li F. F. et al., 2020). A total of 94 antibiotics were found in water bodies and sediments of seven major rivers and four major bay basins in China (Li et al., 2018); 26 antibiotics were detected in surface water of Huangpu River in Shanghai, China (Pan et al., 2020). In addition, antibiotics entering the environment may exert selective pressure on the spread of antibiotic resistance bacteria (ARB) and antibiotic resistance genes (ARGs), which is potentially more harmful than the antibiotics themselves (Zhang et al., 2015; Qiao et al., 2018; Chen Y. et al., 2020).

As a pivotal link in the treatment of industrial and domestic wastewater, wastewater treatment plants (WWTPs) are responsible for the removal of antibiotics from wastewater (Li J. N. et al., 2016; Chen Y. et al., 2020; Xu et al., 2020). However, conventional WWTPs were not specifically designed for the removal of antibiotics (Chen Y. et al., 2020) and antibiotics entering the biological units of WWTPs often change the microbial community structure, affect the stability of the biological treatment units and reduce the treatment efficiency and even cause the collapse of the biological treatment systems (Meng et al., 2017a,b; Zhang Z. H. et al., 2018). In addition, the selective pressure of the WWTPs also promotes horizontal genes transfer (HGT) and vertical genes transfer (VGT), which further diffuse ARGs into the environment (Jia et al., 2017; Chen Y. et al., 2020; Meng et al., 2020). A study of antibiotics and ARGs abundance in a WWTP and its upstream and downstream reaches in Nanjing, China, found that the level of antibiotics in the effluent of the treatment plant was reduced by 90% compared to the raw water and the number of detected antibiotics was reduced from 10 to 5. However, at the level of the ARGs only the abundance of *cmlA* decreased while the abundance of *sul1* and *sul3* was increased in a different degree (Chen Y. et al., 2020). A study of changes in the abundance of ARGs in two WWTPs in Beijing and Kunming, China, further showed that the association between antibiotics and ARGs are not simply direct but that the antibiotic-induced changes in the structure of bacterial communities in the sludge have an impact on ARGs (Xu et al., 2020). In other words, WWTPs are an important sink for antibiotics and an important source of ARGs.

In addition to antibiotic and ARGs contamination in wastewater, the risk of antibiotics and ARGs in organic solid waste such as kitchen waste (Sabri et al., 2020), animal manure (Tong et al., 2022) and residual sludge from WWTPs (Dubey et al., 2021) should not be overlooked. Tong’s study showed that farm swine manure is an important vector for the transmission of ARGs to the surrounding environment, and tigecycline resistance genes *tet(X), tet(X1)*, and *tet(X10)* are prevalent in farms. Farm workers and the surrounding environment are the main recipients of ARGs, sharing at least 90% of their ARGs abundance (Tong et al., 2022); Wang et al.’s (2022c) study noted the abundance of ARGs in residual sludge from a municipal WWTP up to 3.26 × 10^9^ copies/g dry solid; Sahar detected six antibiotics in dewatered sludge from WWTPs with a total concentration of 1,300 ng/g (Dalahmeh et al., 2022). It is estimated that China generates about 40 million tons of kitchen waste (Wang et al., 2022d) and 58 million tons of residual sludge (Wang et al., 2022a) annually, and this figure would be even larger on a global scale (Wang et al., 2022d).

For the treatment of huge amounts of wastewater and organic solid waste, there is no doubt that biological treatment has significant advantages in terms of operating costs. Considering the high organic loadings of the above pollutants, it seems that the use of anaerobic biological treatment is more appropriate than aerobic treatment. Anaerobic biological treatment plays a pivotal role due to the advantages of low operating costs, low sludge production, and biogas recovery (Chen et al., 2008; Cao and Pawlowski, 2012; Rizzo et al., 2013; Zhang et al., 2022c). However, current anaerobic treatment technologies still have limits such as vulnerability to environmental impacts, long conversion time of intermediates fermentation and low organic matter conversion rates (Meng et al., 2017a; Zhao et al., 2017; Huang et al., 2021). In addition, the bactericidal or bacteriostatic properties make it inevitable that antibiotics entering the biological treatment unit will markedly reduce the efficiency of the biological treatment system and even collapse it (Khurana et al., 2021). Therefore, breaking the bottleneck of traditional anaerobic biological treatment technology to further improve the treatment efficiency becomes a challenge. Among the various anaerobic biological treatment enhancement technologies, using electron shuttle is a promising technology. Electron shuttle can reduce the difficulty of electron transfer between electron donor and electron acceptor, improve the transfer efficiency of electron transfer, reduce the activation energy of the reaction and increase the reduction and oxidation rate of pollutants by one to several orders of magnitude (Van der Zee et al., 2003). Although there are some review articles on enhanced anaerobic biological treatment with some electron shuttles (e.g., activated carbon, metal materials), they mainly focus on enhanced anaerobic methanogenesis. To our knowledge, there is no review on enhanced anaerobic biological treatment with electron shuttles to remove antibiotics and resistance genes. Therefore, this paper introduces the development of electronic shuttle technology and the mechanism of action of different electronic shuttles to enhance anaerobic biological treatment, and collates the applications of electronic shuttles in enhancing anaerobic biological treatment to remove antibiotics and ARGs in recent years. Finally, the current problems of this technology and the possible future development directions are discussed. This paper provides a reference for subsequent researchers to continue exploring electron shuttle enhanced anaerobic biotreatment for the removal of environmental pollutants.

## Introduction to electron shuttle

In anaerobic biological treatment systems, the redox process based on extracellular electron transfer (EET) is the main metabolic pathway of microorganisms, and it has been demonstrated that the degradation of pollutants by microorganisms is greatly enhanced with the increase of their EET capacity (Li F. H. et al., 2020). Traditionally, electron transfer in anaerobic systems is dependent on intermediates such as H_2_ and formic acid and such electron transfer mechanisms are called interspecies hydrogen transfer (IHT) or mediated interspecies electron transfer (MIET) (Liu et al., 2018; He et al., 2019), while the discovery of direct interspecies electron transfer (DIET) has provided new insights for enhanced anaerobic biological treatment (Summers et al., 2010). Electron shuttle is a relatively simple and efficient means of direct electron transfer facilitation (Liu et al., 2021d). With its favorable dielectric capacity or through the cyclic transformation of oxidation and reduction states, it is possible for microorganisms to accelerate the degradation of extracellular highly toxic pollutants through electron shuttles and thus reducing the toxic effects on themselves (Zhao Z. S. et al., 2020).

### Natural electron shuttle

Biological components such as cytochrome C, riboflavin, conductive nanowires, and some natural organic substances (e.g., humic substances) can naturally act as electron shuttles to facilitate EET due to their excellent dielectric capacity and unique biochemical properties (Reguera et al., 2005; Sutton and Sposito, 2005; Watanabe et al., 2009; Dang et al., 2016; Faustino et al., 2021). For example, analysis of the structure of the transmembrane protein complex of the extracellular membrane revealed that protein metal reducing AB (MtrAB) can act as an electron transfer channel to transfer intracellular electrons to the extracellular electron receptor by means of MtrC, which is tightly bound to it (Edwards et al., 2020); *Geobacter sulfurreducens* usually achieves long-distance electron transfer by means of its conductive nanowires in direct contact with the electron receptor. Recent studies have shown that the entire nanowire is composed of the cytochrome C protein OmcS, with heme closely arranged on the nanowire acting as an efficient electron transfer agent (Wang F. B. et al., 2019); In addition to acting as a solid electron shuttle, recent studies have found that cytochromes can also participate in the electron transfer process in a solubilized state, with Liu et al.’s (2020c) study showing that *Shewanella oneidensis* MR-1 can release cytochrome C in the solubilized state as an electron shuttle to accelerate electron transfer from cells to Cr(VI) and the reduction of Cr(VI).

However, from the application point of view, it is obvious that the regulation of electron shuttles such as cytochromes and nanowires are not as convenient as the direct addition of exogenous electron shuttles. Therefore, more studies have focused on promoting the anaerobic biological treatment process by adding soluble organic matter such as humic substances and riboflavin. Humic substances are widespread in the environment and are the products of bio-microbial transformation after the death of plants, animals, microorganisms, etc. The composition of humic substances is extremely complex and mainly includes humic acid and fulvic acid (Kelleher and Simpson, 2006), which are generally considered as a class of low molecules composed of aromatic, aliphatic, phenolic, quinone, and N-containing derivatives, covalently bound through C–C, C–O–C, and N–C bonds (Sutton and Sposito, 2005; Kulikova and Perminova, 2021). Its abundant phenolic and quinone moieties play an important role in it. It was noted that quinone groups in humic substances can act as electron acceptors (Cervantes et al., 2001), while phenolic groups have antioxidant activity (Aeschbacher et al., 2012) and can mitigate the growth inhibition of microorganisms by quenching free radicals in an unfavorable environment (Klein et al., 2018). Studies have shown that the addition of humic substances to anaerobic systems can help break the energy barrier between microorganisms and promote electron transfer between different strains to improve anaerobic treatment efficiency (Zheng et al., 2019; Gao et al., 2021). Humic substances have shown significant effects in enhanced biological denitrification (Li M. et al., 2016; Liu et al., 2020b), biological dichlorination (Yuan et al., 2019; Liu C. Y. et al., 2021a), and organic solid waste treatment (Gao et al., 2021). As a cellular secretion, riboflavin has been proved in many studies to be involved in extracellular electron transfer processes (Song et al., 2019; He et al., 2020; Wang J. H. et al., 2020). Liu et al.’s (2020d) study showed that 2 μM riboflavin as an electron shuttle significantly promoted the decolorization efficiency of *Shewanella putrefaciens* CN32 wild-type to the dye acid yellow 36; Ramos-Ruiz et al. (2016) observed that riboflavin increased the reduction efficiency of anaerobic granular sludge system to tellurate by 11 times, and further studies found that the inhibition of anaerobic methanogenic activity by tellurate was mitigated by adding riboflavin to the UASB reactor, with the COD removal rate of the riboflavin-added reactor increasing by 4.6 times higher than that of the reactor without riboflavin addition (Ramos-Ruiz et al., 2017).

### Artificial electron shuttle

#### Liquid electron shuttle

Common liquid electron shuttles include phenazines such as neutral red and quinones represented by sodium 1,2-naphthoquinone-4-sulfonate (NQS), anthraquinone-2-sulfonate (AQS), and anthraquinone-2-6-sulfonate (AQDS).

As a cationic stain, neutral red is commonly used as a histological dye with a high lipid-water partition coefficient of 30 and thus is strongly hydrophobic (Harrington et al., 2015). Properties such as low toxicity and low redox potential (Eo = –525 mV Ag/AgCl) make neutral red promising for applications (Park and Zeikus, 1999). Research has shown that neutral red can reduce the need for cellular oxidation of NADH to produce protons for respiration by driving the reduction of terminal electron acceptors through the reduction process of oxidized methyl naphthoquinone in an anaerobic environment (Harrington et al., 2015).

As mentioned previously, many studies have pointed out that quinone groups in humic substances play an important role in mediating electron transfer. As a class of model quinone compounds, AQS and AQDS can also act as electron shuttles to facilitate the anaerobic process. Usually, after entering anaerobic system, AQDS is first reduced to its hydroquinone state AH_2_QDS by reducing components and then oxidized back to AQDS by oxidizing components (Cai et al., 2021), with the help of multiple cycles of AQDS/AH_2_QDS to promote electron transfer (Yang et al., 2019; Zhou et al., 2021d); Similar to the redox cycle of AQDS, AQS is first reduced to its hydroquinone state AH_2_QS and AHQS, which is later oxidized to AQS (Cai et al., 2021; Xu et al., 2021a).

However, soluble electron shuttles inevitably face the problems of losing and causing secondary contamination with the effluent. Therefore, researchers have recently explored the possibility of loading soluble electron shuttles onto solid-phase carriers such as chitosan (Zhou et al., 2021d), various carbon materials (Atilano-Camino et al., 2020; Wang et al., 2021a), and foam (Lu et al., 2021) to convert the reaction system from homogeneous to multiphase in order to prolong the service life of electron shuttles and reduce possible contamination. Formally, these electron shuttles have been transformed from the soluble state to the solid state.

#### Solid electron shuttle

Solid electron shuttles mainly include carbon-based electron shuttles and metal-based electron shuttles.

Carbon-based materials were first used as adsorbents in water treatment (Gopinath et al., 2021; Zhou et al., 2021b). Recently, many studies have demonstrated that carbon-based materials such as granular activated carbon (GAC) and biochar (BC) can also be used as electron shuttles to effectively facilitate the anaerobic DIET process due to their excellent dielectric capacity and redox activity (Zhao and Zhang, 2019; Deng et al., 2021; Zhang et al., 2022b). For example, Liu T. X. et al. (2021b) used GAC with exogenous hydrogen to enhance the anaerobic reactor to degrade butanol-octanol wastewater generated from coal syngas production, found that the methane production in the GAC/exogenous hydrogen treatment group was significantly enhanced compared to the control group and the relative abundance of *Geobacillus* and *Methanomonas* in the sludge of this group increased rapidly, speculating that GAC could enhance the anaerobic process by stimulating DIET; Liu et al. (2020a) used graphite-modified high-density polyethylene as a carrier to improve the anaerobic integrated floating fixed membrane-activated sludge process and found that the organics degradation rate and methane yield of the reactor with the modified filler were significantly higher than those of the conventional filler reactor and the electron exchange capacity of *Geobacter* and *Methanothrix* were increased by 4.2, 7.3%, respectively. In addition, some studies have shown that the addition of carbon materials into anaerobic systems helps to inhibit the transfer of ARGs among bacteria (Liu et al., 2021c, 2022; Fang et al., 2022).

Furthermore, various carbon nanomaterials such as graphene, graphene oxide (GO), carbon nanotube (CNT), and carbon quantum dot (CD) have been investigated because of their unique size and surface effects and favorable biocompatibility in enhancing anaerobic bioprocessing (Kumar et al., 2021; Deng et al., 2022). Yang et al.’s (2020) study found that the use of CDs enhanced the extracellular electron transfer capacity and metabolism of the model bacterium *Shewanella oneidensis* (*S*. *oneidensis*) MR-1; Li H. Y. et al. (2021) found that the reduction of iron hydroxyl oxide (FeOOH) by *Shewanella putrefaciens* CN32 enhanced with CNT is effective in promoting the TBBPA degradation. After adding CNT to the anaerobic system, the Fe(II) concentration in the system was 235.5% of that in the control group without CNT, and the TBBPA removal rate increased from 20.5 to 87.1%, which was mainly attributed to the fact that CNT as an efficient electron shuttle facilitated the reduction of FeOOH by CN32 and thus produced more Fe(II) for the reduction and degradation of TBBPA (Li H. Y. et al., 2021). However, it should be noted that due to the relatively high cost of these nanomaterials, research on them is currently limited to the laboratory stage. The cost of the method is something we have to take into account when the electron shuttle technology are applied in reality engineering.

Metal-based electron shuttles include zero valent metals and metal oxides. Current studies on zero valent metal electron shuttles have focused on zero valent iron (ZVI) and nano zero valent iron (nZVI) (Jadhav et al., 2021; Shi et al., 2021; Ye et al., 2021), with a few remaining metals such as cobalt (Abdelwahab et al., 2021), aluminum (Calabro et al., 2021), etc. Zhu et al.’s (2020) study pointed out that ZVI promotes anaerobic biological treatment mainly through two pathways: (1) ZVI enriches hydrotropic methanogenic bacteria to reduce the H_2_ partial pressure in the system to promote acidification (i.e., improve IHT); (2) acts as a conductive medium and stimulates the secretion of extracellular polymeric substances (EPS) to promote direct electron exchange between microorganisms and establish direct interspecies electron transfer (i.e., promote DIET). In this process, the particle size and dose of ZVI dosed will affect the anaerobic treatment effect (Xu et al., 2021b).

**FIGURE 1:**
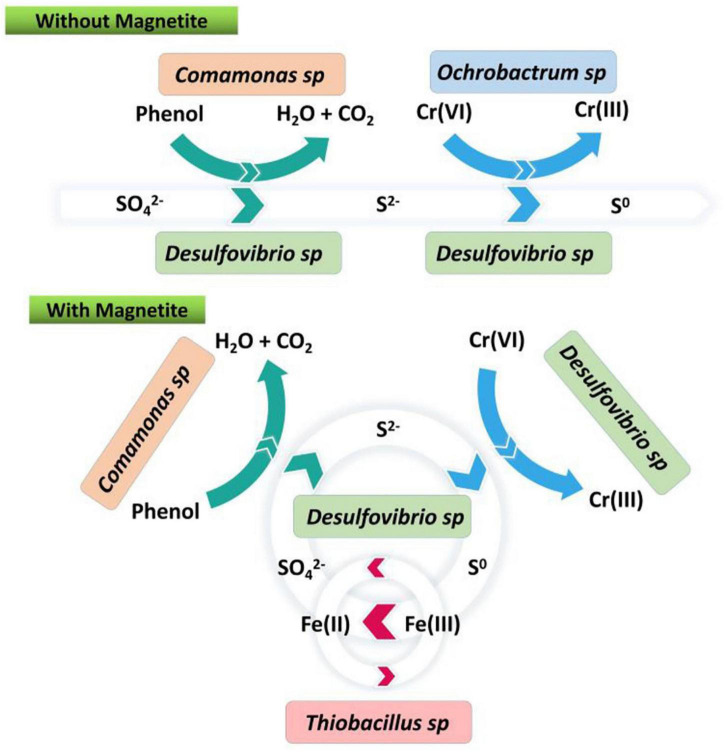
Microbial sulfur cycle for phenol degradation coupled with Cr(VI) reduction via the potential Fe(III)/Fe(II) transformation (Zhao Z. Q. et al., 2020).

In the case of metal oxides, in addition to acting as strong conductive media to establish DIET processes, they often provide efficient electron transfer by cycling metal ions between high and low valence states (Xu et al., 2022). Coupling this process with other forms of cyclic processes can greatly improve the efficiency of anaerobic treatment. For example, Yang et al. (2019) put Fe_2_O_3_ and AQDS together in an anaerobic system to build a double cycle of Fe(III)/Fe(II) and AQDS/AH_2_QDS to achieve a nitrogen removal efficiency of 82.6%; further, continuous denitrification by intermittent aeration into the anaerobic system with the help of Fe(III)/Fe(II) redox cycle increased the total nitrogen removal up to 98.5% (Yang et al., 2021c); redox cycle of Fe(III)/Fe(II) can also be used in the efficient degradation of various environmental pollutants. Li et al. (2022) introduced magnetite into the anaerobic sulfate reduction system and found that the addition of magnetite enriched the sulfur disproportionation microorganism *Desulforvibrio aminophilus*, creating an Fe-sulfur cycle by coupling the process of sulfide oxidation to singlet sulfur with Fe(III) reduction. The benzoate degradation rate was increased from 56.3 to 77.1% (Li et al., 2022); similarly, Zhao Z. Q. et al. (2020) introduced magnetite into the anaerobic sulfate reduction system and increased phenol removal from 53.1 to 95.5% through an Fe-sulfur cycle (Figure 1). Jin introduced Fe(OH)_3_ into the sulfate-containing azo dye acid orange 7 (AO7) wastewater and found that the AO7 degradation products 1A2N and its reduced state 1I2NQ could construct an electronic cycle with Fe(III)/Fe(II), which consequently promoted azo bond breaking. The COD removal and decolorization efficiency of the Fe(OH)_3_-dosed group were 61.7 and 32.0% higher than the control group, respectively. Besides, the concentration of cytochrome C and the conductivity of the suspended sludge were also 3.2 and 2.1 times higher than the control group (Li et al., 2017).

In addition, among the aforementioned carbon-based electron shuttles and metal-based electron shuttles, adding carbon nanomaterials alone often faces difficulties in recycling, while adding metal nanomaterials alone may lead to material agglomeration causing a significant decrease in the utilization efficiency (Kassab et al., 2020; Wang et al., 2021b). For both recyclability and utilization efficiency, combining micron or nanoscale metal materials with conventional scale carbon materials to construct metal-carbon composite electron shuttles is also a common strategy (Wan et al., 2021; Xu et al., 2021c; Zhang and Wang, 2021; Che et al., 2022).

## Application of electron shuttle in enhanced anaerobic treatment of antibiotics and ARGs

### Wastewater treatment

In Table 1, we list some research on removal of antibiotics and ARGs by enhanced anaerobic biological treatment with electron shuttles. Zhou et al. (2018) explored the effect of AQDS and riboflavin as electron shuttles to promote the degradation of antibiotic sulfamethoxazole by MR-1, showing that the addition of AQDS and riboflavin increased the removal rate of sulfamethoxazole from 38.5 to 70.7% and 95.3%, respectively. Moreover, the degradation rate of sulfamethoxazole gradually increased with the increase of iron reduction intensity (Zhou et al., 2018). Liu et al. (2022) found that the input of biogenic carbon into the anaerobic system was effective in mitigating the horizontal gene transfer effect of Cu(II) on ARGs between *Escherichia coli* DH5α and *E. coli* HB101 through the regulation of three globally regulated genes (*korA, korB, trbA*). Xiao et al.’s (2022) study pointed out that the removal of chloramphenicol by microbial fuel cell containing the electroactive bacteria *Geobacter metallireducens* could increase from about 50 to 100% after addition of Fe_3_O_4_ and MnO_2_ nanoparticles, respectively. Mechanistic analysis showed that Fe_3_O_4_ nanoparticles mainly enhanced the expression of C-type cytochromes and ethanol dehydrogenase, while MnO_2_ nanoparticles mainly enhanced the expression of type IV pili and pyruvate dehydrogenase, the expression of NADH-quinone oxidoreductase was increased by both nanoparticles (Xiao et al., 2022).

**TABLE 1:** Summary of research on removal of antibiotics and ARGs by enhanced anaerobic biological treatment with electron shuttles.

Digestion substrate	Target contaminants	Electron shuttle	Dosage	Removal rate	References
Food waste, chicken manure	*tetA, tetB, tetM, tetW, tetQ, tetO, tetX, sul1, sul2, cmlA, floR*	AC	15g/L	–	Zhang J. X. et al., 2018
Food waste	*tetA, tetB, tetM, tetW, tetQ, tetO, tetX, sul1, sul2, cmlA, floR*	AC	15g/L	–	Zhang J. X. et al., 2017
Swine wastewater	ARGs	dewatered swine manure-derived BC	20 g/L	99.99%	Wang et al., 2022b
Swine manure	ARGs	BC	5–20%	40.6–51.7%	Yang et al., 2021b
Swine wastewater	sulfadiazine, sulfamethazine	pomelo peel derived BC	0.5 g/L	74.1%, 80.1%	Cheng et al., 2021
Chicken manure	ARGs	AC	–	87–95%	Zhang et al., 2019c
Synthetic wastewater	ciprofloxacin	CNT and magnetic CNT	0.1 g/L	99%	Silva et al., 2021b
Chicken manure	*tetW, tetO, tetC, tetG, ermB, sul1* and *sul2*	nZVI	600 mg/L	44.7–81.3%	Qiu et al., 2022
Swine manure	ARGs	ZVI	75 mmol	25%	Zhang J. Y. et al., 2021
Synthetic wastewater	chloramphenicol	nZVI	1 g/L	99.2%	Li J. H. et al., 2021
Synthetic wastewater	tet ARGs	ZVI	5 g/L	95% (sludge), 72% (effluent)	Xu et al., 2021e
Synthetic wastewater	tet ARGs	ZVI	5 g/L	0.75–1.88 log (intracellular), 0.67–2.08 log (extracellular)	Xu et al., 2021d
Sewage sludge	sulfamethoxazole, sulfamerazine, tetracycline, roxithromycin	ZVI	1,000 mg/L	97.39%, 74.54%, 78.61%, 56.58%	Zhou et al., 2021a
Swine manure	sulfadiazine	Fe-0	5 g/L	86.8%	Huang H. N. et al., 2019
Dewatered sludge and waste activated sludge	*aac(6′)-IB*	Fe_3_O_4_ NPs, nZVI	0.5 g/L, 1.0 g/L	96.50%, 95.83%	Xiang et al., 2019
Food waste	ARGs	nZVI	2 g/L	86.64%	Wang P. et al., 2019
Cattle manure	ARGs	nZVI	160 mg/L	75%	Ma et al., 2019
Synthetic wastewater	tetracycline	nZVI	0.50 g/gVS	>70%	Pan et al., 2019
Synthetic wastewater	tetracycline	Fe_3_O_4_	5 g/L	99.9%	Zhao Z. S. et al., 2020
Secondary sludge and dewatered sludge	ARGs	Fe_3_O_4_ NPs, nZVI	0.5 g/L, 4.0g/L	70.73%, 62.69%	Zhang et al., 2020
Synthetic wastewater	ciprofloxacin	nZVI/AC	0.56 g/gVS	72.41%	Zhou et al., 2021c
Synthetic wastewater	tetracycline	nZVI/GAC	1,000 mg/L, 1,200 mg/L	81.5%	Zhang Z. H. et al., 2018

Nascimento added AQDS to a UASB reactor with a low hydraulic retention time (7.4 h) to remove sulfamethoxazole and methomyl, increasing their removal from 6 to 70% (do Nascimento et al., 2021); Silva used CNT to enhance the removal of ciprofloxacin from anaerobic granular sludge and found that the removal of ciprofloxacin could reach 99% with the addition of 0.1 g/L of CNT. Further analysis of effluent biotoxicity showed that enhanced anaerobic treatment with CNT resulted in a detoxification rate of approximately 46% for *Vibrio fischeri* (Silva et al., 2021b). Li J. H. et al. (2021) study found that adding 1 g/L of nZVI to the anaerobic system significantly increased the removal of chloramphenicol from 46.5 to 99.2%, mainly because the addition of nZVI enabled the enrichment of dechlorination-related bacteria and functional bacteria associated with refractory pollutants. In addition, on one hand, the high concentration of nZVI acted as a chloramphenicol adsorbent to reduce the antibiotic pressure in the system, and on the other hand made a reduction in the potential hosts of ARGs, leading to a simultaneous reduction of antibiotics and ARGs (Li J. H. et al., 2021). Contrasting with the enhanced anaerobic treatment of chloramphenicol with electrically assisted, although 89.7% reduction of antibiotics was achieved, the abundance of ARGs in the system was significantly higher (Guo et al., 2019); Xu’s study also indicated that the mechanism of ARGs reduction by ZVI in wastewater is mainly through the reduction of their potential hosts and inhibition of horizontal and vertical gene transfer processes of ARGs, including activation of the antioxidant system of specific bacteria, blocking their efflux pump mechanism and energy metabolic conversion. It should be noted that the reduction of ARGs was not significantly affected by ZVI dosing at too high a concentration (20 g/L), which may be due to the iron oxidative damage induced by high ZVI dosing (Xu et al., 2021e,d). Zhao Z. S. et al. (2020) enhanced the removal of tetracycline by adding Fe_3_O_4_ to the anaerobic system. On the one hand, Fe_3_O_4_ can adsorb tetracycline in the aqueous phase to provide more effective biodegradation conditions, and on the other hand, the introduction of Fe_3_O_4_ helps to establish the DIET process. Tetracycline removal was increased by 7.3% under glucose/tetracycline co-digestion conditions compared to the group without Fe_3_O_4_ addition, while tetracycline removal was increased by 40.4% under tetracycline digestion alone (Zhao Z. S. et al., 2020). Zhang Z. H. et al. (2018) injected GAC/nZVI into the EGSB reactor to enhance anaerobic biological treatment for tetracycline removal and found that the input of GAC/nZVI led to a significant increase in EPS secretion and electrical conductivity of the sludge. The COD and TOC removal rates of the anaerobic system were increased by 12.1 and 10.3%, respectively, after the addition of the electron shuttle, and the loss of the electron shuttle was only 5.4% after 34 days of operation (Zhang Z. H. et al., 2018); similarly, Zhou et al. (2021c) enhanced the anaerobic biological system with AC/nZVI for the removal of ciprofloxacin. Compared with the blank group, the removal rate of ciprofloxacin in the AC/nZVI group increased from 22.61 to 72.41%, and the volatile fatty acid yield increased by 173.7%. The abundance of microorganisms associated with hydrolysis, acid production and ciprofloxacin degradation were significantly increased after AC/nZVI dosing (Zhou et al., 2021c).

Farm wastewater often contains high abundance of antibiotics and ARGs. Wang added biochar made from dewatered swine manure to swine manure wastewater, which significantly shortened the methanogenic inhibition period and reduced the abundance of total ARGs in the effluent by nearly four logs (9.2 × 10^8^–9.1 × 10^4^) with a reduction of MGEs by 74.8% (Wang et al., 2022b); Cheng et al.’s (2021) study showed that the addition of 0.5 g/L teak peel biochar to an anaerobic membrane bioreactor increased the removal rate of sulfadiazine and sulfamethoxazole by more than 30% while alleviating membrane contamination caused by sulfonamide antibiotics; Yao et al. (2021) used ZVI to enhance the anaerobic treatment of livestock wastewater containing loxarsine and found that the addition of 1 g/L ZVI reduced the methanogenic inhibition of loxarsine by 80.7%.

### Organic solid waste treatment

High concentrations of antibiotics present in residual sludge not only inhibit the methanogenic process of anaerobic digestion of sludge, but also pose an environmental risk (Ni et al., 2020). Zhou et al. (2021a) used ZVI powder to enhance anaerobic digestion of medium-temperature sludge and found that the anaerobic reactor could remove sulfamidine, sulfamethoxazole, tetracycline, and roxithromycin up to 97.39, 74.54, 78.61, and 56.58%, respectively. After the application of 1 g/L of ZVI, the abundance of *AAC (6′)-IB-CR* and *tetB* was significantly reduced compared to the control group without ZVI (Zhou et al., 2021a). Xiang et al.’s (2019) study showed that enhanced anaerobic digestion of municipal sludge using nZVI contributed to the removal of *ermA* and *ermT* (Xiang et al., 2019); Zhang et al. (2020) used Fe_3_O_4_ nanoparticles and nZVI for enhanced anaerobic digestion of municipal sludge and found that the removal of total ARGs by both iron nanoparticles was 70.73 and 62.69%, respectively. There was a significant advantage of iron-based nanomaterials for *blaOXA* reduction, which was mainly due to the reduction of its potential hosts (Zhang et al., 2020). However, it is necessary to point out that some metal nanomaterials not only do not contribute to reduce ARGs, but also pose the risk of dispersal of ARGs. For example, Huang H. N. et al. (2019) and Zhang Y. R. et al. (2021) study pointed out that nano-metal oxides such as CuO, ZnO, and Al_2_O_3_ present in the residual sludge may increase the abundance of MGEs and thus aggravate the dispersion of ARGs during anaerobic digestion.

Treating livestock manure generated on farms using aerobic/aerobic composting or co-digestion with sludge is a common response today, but conventional treatment methods are not effective in removing antibiotics and ARGs (Chen J. F. et al., 2020; Li H. Y. et al., 2020; Katada et al., 2021). Yang et al. (2021b) used biochar to enhance swine manure anaerobic composting and found that the addition of biochar helped to enrich DIET microorganisms, and the removal of *parC, tetX, blaCTX-M, blaTEM*, and *ermF* in the biochar treatment group exceeded 85% with 25% higher methane yield; Zhang J. Y. et al. (2017) study noted that the addition of 500 mg/L of GO removed 40.2% of ARGs, but GO at 5 and 100 mg/L may deteriorate the removal of ARGs (3.7, 23.9%, respectively); Zhang et al. (2019d) introduced GO into swine manure with high concentrations of Cu for anaerobic digestion and found that GO helped to reduce the abundance of ARGs and MGEs in the anaerobic digestion system and the removal effect of low concentration of GO (100 mg/L) was better than that of high concentration of GO (800 mg/L); Wang Q. Z. et al. (2020) used ZVI to enhance the removal of ARGs from swine manure anaerobic compost and suggested that ZVI could also reduce the environmental risk of ARGs by reducing the abundance of MGEs in swine manure. Qiu et al. (2022) used nZVI to enhance anaerobic composting of chicken manure and found that nZVI dosing decreased the abundance of *tetW, tetO, tetC, tetG, ermB, sul1*, and *sul2* by 66.3, 81.3, 76.8, 59.7, 44.7, 74.4, and 67.2%, respectively. While the abundance of all ARGs except *sul1* and *sul2* increased to varying degrees in the control group without ZVI (Qiu et al., 2022); Huang W. W. et al. (2019b) found that the addition of 5.0 g/L of ZVI during anaerobic composting of swine manure increased the removal of sulfadiazine by 86.8% and total solids by 26.4%. Ma et al.’s (2019) study showed that the ARGs removal rate could be increased by 75% with nZVI injection of 160 mg/L during anaerobic digestion of cattle manure compared to the control group without nZVI; Zhang et al.’s (2019b) study found that Fe_3_O_4_ applied to enhanced anaerobic digestion of swine manure could effectively reduce tetG, *tetM* and *tetX*, with *tetX* removal rate reaching 70.2%, while no Fe_3_O_4_ control group showed varying degrees of increase in these three ARGs. In addition, Zhang combined the microwave pretreatment process with activated carbon enhanced anaerobic digestion to synergistically promote anaerobic digestion of chicken manure to remove ARGs, which achieved 87–95% removal of ARGs, significantly better than the control group without microwave and activated carbon treatment (34–58%) (Zhang et al., 2019c).

In terms of kitchen waste treatment, Zhang J. X. et al. (2017) study pointed out that the effect of AC addition on the removal of tetracycline ARGs (e.g., *tetA, tetM, tetW, tetO, tetQ*, and *tetX*) during anaerobic fermentation of kitchen waste was better than without AC. Moreover, the addition of AC resulted in a more stable anaerobic digestion system. Further comparison of the effects of AC on the three modes of kitchen waste digestion alone, kitchen waste-chicken manure co-digestion, and kitchen waste-residual sludge co-digestion revealed that the removal of ARGs in the experimental group with AC addition was better than the corresponding control group without AC addition (Zhang J. X. et al., 2018); Wang et al. (2021c) compared the influence of graphite, graphene and GO on the co-digestion of sludge and kitchen waste and the abundance of ARGs, finding that compared with graphite and GO, graphene had significant advantages on the removal of blaOXA-1, macrolide resistance genes (*ermF* and *ermB*) and some tetracycline resistance genes (*tetQ* and *tetX*), with the removal rates of 89.90, 57.75, 96.11, 95.22, and 88.76%, respectively. While GO showed a significant removal advantage for sulfonamide resistance genes (*sul1, sul2*) and some tetracycline resistance genes (*tetM, tetO*, and *tetW*) with the removal rates of 84.61, 76.12, 87.79, 90.50, and 75.66%, respectively. Furthermore, GO significantly reduced the abundance of the mobile gene element intl1 (86.17%), which helped to control the horizontal gene transfer of ARGs (Wang et al., 2021c); Wang achieved 86.64% removal of ARGs by feeding 2 g/L of ZVI into the anaerobic digestion system of kitchen waste at high temperature (55°C) (Wang P. et al., 2019).

## Mechanisms of electron shuttle enhanced anaerobic treatment of antibiotics and ARGs

### Enhanced antibiotic treatment

**FIGURE 2:**
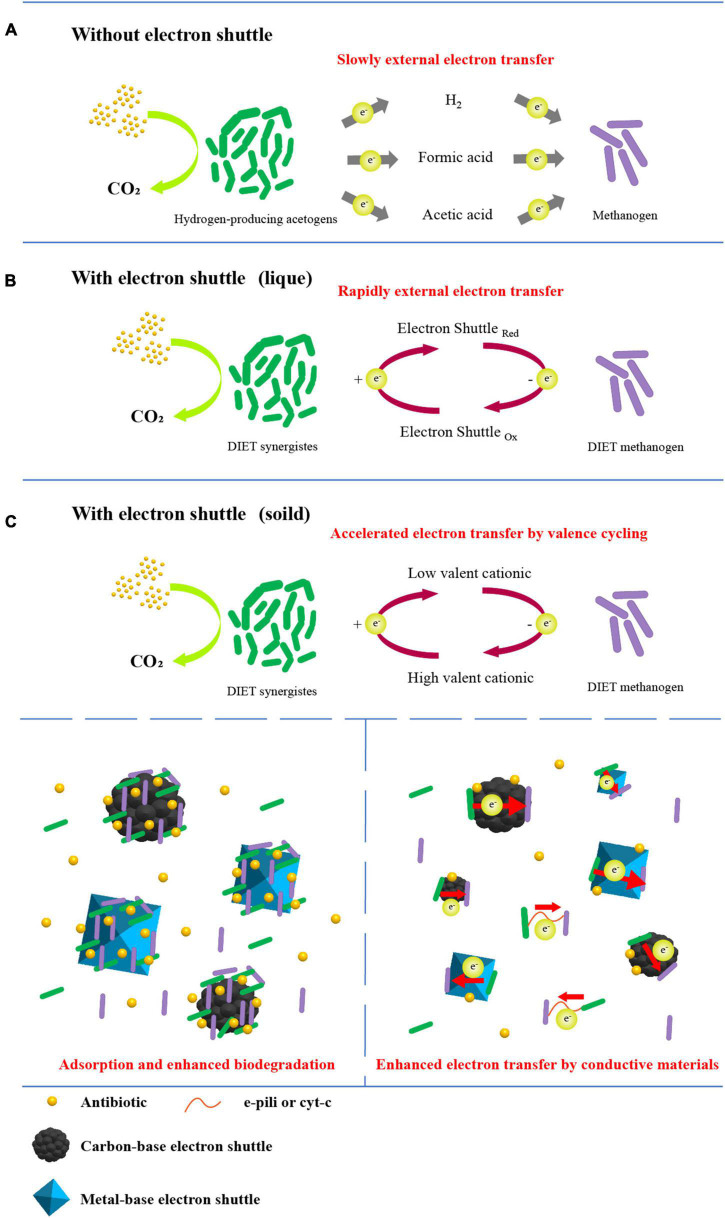
Possible mechanisms of anaerobic treatment of antibiotics **(A)** without electron shuttle, **(B)** enhanced by liquid electron shuttle, **(C)** enhanced by solid electron shuttle.

Figure 2 summarizes the mechanism of liquid or solid electron shuttles enhanced anaerobic treatment of antibiotics. For soluble electron shuttles, the enhancement of electron transfer efficiency between microorganisms and antibiotics is their main pathway of enhancing antibiotic removal. For example, riboflavin, a natural class of electron shuttles, can account for up to 75% of the total microbial electron transfer process (Zhou et al., 2018); Synthetic AQS and AQDS as model quinone species can significantly enhance microbial extracellular electron transfer with the help of the cycle of their oxidation and reduction states (do Nascimento et al., 2021).

In contrast, the pathways of solid electron shuttles for enhanced antibiotic removal are more abundant. Mainly, (1) carbon-based and metal-based materials cast into anaerobic systems, especially in wastewater treatment, serve as good carriers for microbial growth and can significantly improve microbial growth activity (Zhao Z. S. et al., 2020; Yang et al., 2021a); (2) antibiotics in the aqueous phase are adsorbed onto the surface of the electron shuttle by adsorption (Zhao Z. S. et al., 2020; Cheng et al., 2021), due to their excellent electrical conductivity acting as an microbial-antibiotic “electron bridge” to enhance the electron transfer process (Zhang Z. H. et al., 2018; Zhou et al., 2021c); (3) the pili and cytochrome C of bacteria are important mediators of the microbial extracellular electron transfer process. It has been pointed out that metal oxides (e.g., Fe_3_O_4_, MnO_2_) help to stimulate the expression of relevant functional genes and promote them with different effects (Xiao et al., 2022). In some cases Fe_3_O_4_ can replace cytochrome C to play an extracellular electron transfer role (Jin et al., 2019); (4) for some metal-based electron shuttles, efficient electron transfer can be achieved with the help of cycling of high and low valence of dissolved metal ions, such as Mn(IV)/Mn(II) cycle and Fe(III)/Fe(II) cycle (Xu et al., 2022).

In addition, the introduction of either soluble or solid shuttles into anaerobic biological systems enriched bacteria with DIET capability resulting in a significant enhancement of extracellular electron transfer efficiency (Zhao Z. S. et al., 2020; do Nascimento et al., 2021).

### Enhanced removal of ARGs

**FIGURE 3:**
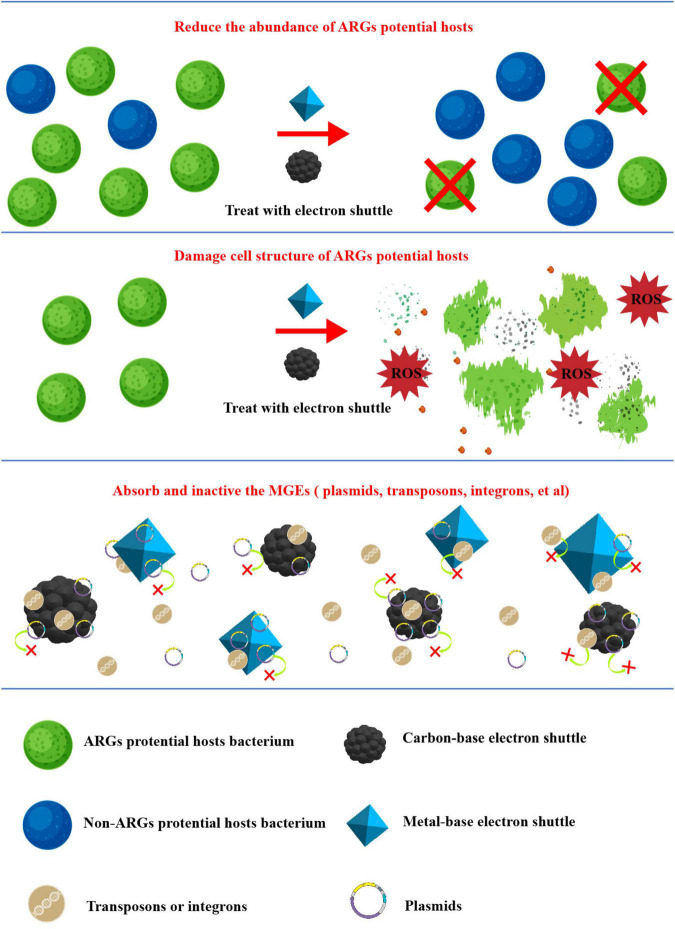
Possible mechanisms of electron shuttle enhanced anaerobic treatment of ARGs.

The mechanism of liquid or solid electron shuttles enhanced anaerobic treatment of ARGs are summarized in Figure 3. ARGs migrate and disperse into the environment through two pathways, HGT and VGT (Zhang et al., 2022a). From the perspective of inhibiting VGT, on the one hand, electron shuttles introduced into anaerobic biological systems can reshape the community structure and reduce the abundance of potential hosts of ARGs (Zhang et al., 2019a, 2020; Xu et al., 2021e); On the other hand, nanoscale electron shuttles, especially metal-based electron shuttles, can damage cell structure and lead to the release of intracellular reactive oxygen species and alter cell membrane permeability to interfere with cell growth and metabolism and finally inhibiting the growth and even death of the host cells involved (Su et al., 2019; Zhang J. Y. et al., 2021; Qiu et al., 2022).

From the perspective of inhibiting HGT, ARGs can spread between different strains with the help of Mobile Genetic Elements (MGEs) such as plasmids, transposons, and integrons. Studies have pointed out that biochar and nanometallic materials can inhibit the spread of ARGs in the environment by adsorption, causing intra-plasmid condensation clustering and damaging the plasmid structure (Su et al., 2019; Fang et al., 2022); class 1 integron-integrase gene (*intI1*) is usually used as an indicator gene for MGEs. Many studies have shown a significant correlation between *intI1* and changes in the abundance of ARGs during anaerobic bioprocessing of ARGs by various types of electron shuttles (Li J. H. et al., 2021; Yang et al., 2021b; Qiu et al., 2022), i.e., electron shuttles can control the abundance of ARGs by reducing MGEs; it has also been suggested that the adsorption by electron shuttles decreases the mobility of microorganisms in anaerobic systems and thus reduce the mobility of microorganisms in the anaerobic system, which in turn limits the possibility of HGT and thus limits the enrichment of ARGs (Zhang J. X. et al., 2017).

## Conclusion and perspectives

For the current widespread pollution of antibiotics and ARGs in the environment, enhanced anaerobic biological treatment with electron shuttles for the removal of antibiotics and ARGs is a promising technology. The electron shuttle achieves efficient degradation of pollutants by (1) using redox cycling process or excellent electrical conductivity or (2) enriching microorganisms capability of DIET to accelerate extracellular electron transfer.

As soluble electron shuttles, natural or artificial components such as riboflavin, AQS and AQDS were used to enhance anaerobic biological treatment. Compared with soluble electron shuttle, solid electron shuttle such as carbon-based or metal-based materials overcomes the disadvantages of losing and causing secondary contamination in effluent and therefore being more widely applied. With the development of nanotechnology, increasing number of nanomaterials such as carbon nanotubes, graphene, graphene oxide, and nanometallic particles are also used as electron shuttles in enhanced anaerobic biological treatment. In the awareness of recyclability and utilization efficiency, a growing number of researchers now tend combining carbon-based materials with nano metal materials to prepare carbon-metal composite electron shuttles.

Numerous studies have shown that the mechanisms of antibiotic removal by electron shuttles enhanced anaerobic systems are: (1) adsorbing antibiotics from the aqueous phase and providing sites for their interaction with microorganisms; (2) increasing the metabolic activity of microorganisms; and (3) facilitating electron transfer between microorganisms and antibiotics through valence cycling or “electron bridges.” For ARGs, the mechanisms of their removal by electron shuttle-enhanced anaerobic systems are (1) modulation of microbial communities to reduce the potential hosts of ARGs, (2) damage to the cellular structure of ARGs hosts, and (3) adsorption and deactivation of MGEs to weaken HGT of ARGs.

In the author’s opinion, there are several shortcomings in the current research in this field or directions that can continue to be explored in the future, as follows.

1. Although nanomaterials are increasingly used as electron shuttles in enhanced anaerobic biological treatment processes, there are few studies on the environmental hazards caused by nanomaterials entering the environment (Silva et al., 2021a). Therefore, more research is needed on the environmental toxicology and ecological risks of nano-based electron shuttles.
2. Carbon-based electron shuttles have drawn attention due to the biocompatibility and excellent ability to mediate electron transfer. Considerable studies have confirmed the significant effect of various carbon nanomaterials on promoting anaerobic processes. However, considering their high preparation cost, it is difficult to apply them to large scale practical applications. Cost-effective and more efficient carbon-based electron shuttles through interfacial modulation and various advanced means is a possible research development direction.
3. Presently, the field of anaerobic biological treatment has been challenged not only by various types of traditionally refractory organic compounds such as PAHs, phenols, PCBs, but also by emerging pollutants such as endocrine disruptors, microplastics, pharmaceuticals and personal care products. In contrast to studies on solid organic waste, current research on wastewater often focuses on anaerobic biological treatment of a specific pollutant, whereas multiple pollutants often coexist in actual wastewater. For example, microplastics have been noted to enhance the propagation of intracellular and extracellular ARGs in municipal wastewater (Cheng et al., 2022). The multi media micro-interface behavior and pollutant removal mechanisms of electron shuttles-microorganisms-pollutants in complex environments remain to be further explored.

## Author contributions

YD: preparing original draft. KZ, JZ, XL, and CH: reviewing. ZW: preparing original draft and reviewing and editing. All authors contributed to the article and approved the submitted version.

## Abbreviations

ARB: antibiotic resistance bacteria
ARGs: antibiotic resistance genes
WWTPs: wastewater treatment plants
HGT: horizontal genes transfer
VGT: vertical genes transfer
EET: external electron transfer
DIET: direct interspecies electron transfer
IHT: interspecies hydrogen transfer
MIET: mediated interspecies electron transfer
GAC: granular activated carbon
NQS: sodium 1,2-naphthoquinone-4-sulfonate
AQS: anthraquinone-2-sulfonate
AQDS: anthraquinone-2-6-sulfonate
GO: graphene oxide
CNT: carbon nanotube
CD: carbon quantum dot
ZVI: zero valent iron
nZVI: nano zero valent iron
EPS: extracellular polymeric substances.
